# Low-loss and polarization insensitive 32 × 4 optical switch for ROADM applications

**DOI:** 10.1038/s41377-024-01456-8

**Published:** 2024-04-24

**Authors:** Xiaotian Zhu, Xiang Wang, Yanlu Huang, Liyan Wu, Chunfei Zhao, Mingzhu Xiao, Luyi Wang, Roy Davidson, Yanni Ou, Brent E. Little, Sai T. Chu

**Affiliations:** 1 Department of Physics, City University of Hong Kong, Tat Chee Avenue, Hong Kong, China; 2 QXP Technologies Inc., NO. 15th Shanglinyuan 1st Road, Xi’an, Shaanxi 710119 China; 3 State Key Laboratory of Information of Photonics and Optical Communications, School of Electronic Engineering, Beijing University of Posts and Telecommunications, Beijing, 100876 China

**Keywords:** Fibre optics and optical communications, Integrated optics

## Abstract

Integrated switches play a crucial role in the development of reconfigurable optical add-drop multiplexers (ROADMs) that have greater flexibility and compactness, ultimately leading to robust single-chip solutions. Despite decades of research on switches with various structures and platforms, achieving a balance between dense integration, low insertion loss (IL), and polarization-dependent loss (PDL) remains a significant challenge. In this paper, we propose and demonstrate a 32 × 4 optical switch using high-index doped silica glass (HDSG) for ROADM applications. This switch is designed to route any of the 32 inputs to the express ports or drop any channels from 32 inputs to the target 4 drop ports or add any of the 4 ports to any of the 32 express channels. The switch comprises 188 Mach-Zehnder Interferometer (MZI) type switch elements, 88 optical vias for the 44 optical bridges, and 618 waveguide-waveguide crossings with three-dimensional (3D) structures. At 1550 nm, the fiber-to-fiber loss for each express channel is below 2 dB, and across the C and L bands, below 3 dB. For each input channel to all 4 drop/add channels at 1550 nm, the loss is less than 3.5 dB and less than 5 dB across the C and L bands. The PDLs for all express and input channels to the 4 drop/add channels are below 0.3 dB over the C band, and the crosstalk is under −50 dB for both the C and L bands.

## Introduction

The surge in 5G, virtual/augmented reality (VR/AR), artificial intelligence (AI), big data, and other advanced digital technologies has dramatically increased the demand for higher-capacity, more flexible, and stable optical networks. All-optical networks based on ROADMs have emerged as a promising solution, enabling the routing of single or multiple channels to optimize traffic dynamically without altering the network infrastructure^1–4^. Their advantages, including remote operation, reconfiguration, and dynamic software-driven, position them as key components in elastic optical networks (EONs)^5,6^.

Three of the most popular ROADMs technologies are: wavelength blocker (WBs), planar Lightwave circuits (PLCs), and wavelength selective switch (WSSs)^7–9^. Presently, micro-electromechanical systems (MEMS) mirrors, including digital light processing (DLP), liquid crystal (LC), and liquid crystal on silicon (LCoS), predominantly serve as WSSs due to their colorless nature, moderately low loss, and flexible wavelength grid. However, their bulk optic components prevent this kind of switch from being compact and cost-effective.

Integrated photonics paves the way for more compact and robust switches, facilitating the transition of ROADMs towards node-on-a-blade^10–12^. Two well-known integrated optical switch elements are micro-ring resonators (MRRs) and MZIs^13–17^. The MRR-type switches are wavelength selective, but it is also very sensitive to temperature variations. The temperature of the device needs to be stabilized and fine-tuning of the wavelengths is needed especially if multi-elements are cascaded when it is put into use. Additionally, because the MRR is an infinite response filter, excess dispersion is introduced to the signal that cannot be neglected. Furthermore, MRRs have a limited wavelength range due to their smaller Free Spectral Ranges (FSR) and are typically highly polarization-dependent. In contrast, MZI-type switches are less wavelength dependent and can eliminate the need for complex tuning and stabilization, if they can be combined with wavelength division multiplexing (WDM) components, making them preferable for ROADM applications where numerous switches are required. Recent years have witnessed the boost of the integrated optical switch based on various platforms and techniques, such as silicon MEMS optical switches^18–21^, silicon thermo-optical (TO) switches^22–25^, silicon (Si) electro-optical (EO) switches^26,27^, silicon nitride (SiN) switches^28,29^, hybrid Si and SiN switches^30,31^. Some of these demonstrations achieved high port counts (up to 128) and fast response times. However, for practical deployment of ROADM, considerations such as low IL, low PDL, and low crosstalk are crucial.

High IL reduces the loss budget of the transmission link, limiting its reach. PDL’s impact is significant in the transmission system since the linear accumulated PDL will cause optical signal-to-noise ratio (OSNR) penalties or restrict the implementation of advanced communication technologies like the dual-polarization coherent communication systems^32–35^. However, most of the reported integrated switches suffer from high IL, including both high fiber-to-waveguide coupling loss (typically 3 to 6 dB/facet) and high on-chip loss (5 to 15 dB depending on the port count)^36–38^. Moreover, few reports detail their PDL performance, as most platforms support only one polarization or exhibit high polarization dependence.

The HDSG platform employed in this work features an adjustable waveguide index contrast of 10% to 20%, which is lower than the Si and SiN waveguides but higher than the low index contrast silica waveguides^39^. It can strike a balance between fiber coupling loss, propagation loss and PDL, while maintaining high integration density. Additionally, the deposition of the film using low-temperature plasma-enhanced chemical vapor deposition (PECVD) enables the waveguides to exhibit even lower birefringence. With the assistance of monolithically integrated spot-size converters, the coupling loss from standard single-mode-fiber (SMF) with a mode field diameter of 10.4 μm into waveguides with 2 μm by 2 μm mode fields is lower than 0.4 dB/facet and PDL below 0.07 dB across the C and L bands. These attributes of low loss and low PDL make the platform promising for the integration of switch devices.

In this paper, we demonstrate a complementary metal-oxide-semiconductor (CMOS) compatible HDSG platform based 32 × 4 optical switch for ROADM applications. The switch comprises 188 MZI-type switch elements, 88 optical vias, and 618 waveguide-waveguide crossings distributed across two vertical layers using a 3D integration process. The measured fiber-to-fiber losses on express and drop channels of the fully optical and electronic packaged device are within 3 dB and below 5 dB for 32 channels, respectively, within C and L bands. The PDL of the device is below 0.3 dB in the C band and the crosstalk of all 32 input channels is less than -50 dB for express cases and -40 dB for drop/add cases. Thirty-two variable optical attenuators (VOAs) are also incorporated and the PDL is lower than 1 dB within the C band at 10 dB VOA attenuation.

## Results

### Waveguide design and switch architecture

All waveguide circuit elements are implemented in the CMOS-compatible low-loss HDSG platform, with a waveguide core index of 1.60, and a cladding index of 1.45, resulting in an index-contrast of approximately 10%. This moderate index contrast is chosen to enable significant device size reduction compared to conventional low-index PLCs, while still maintaining superior fiber coupling efficiency and fabrication sensitivity than the very high index contrast platforms such as Si and SiN^39^. The bus waveguides have dimensions of 2 μm in height, and roughly 2 μm in width, while the bending radius is 100 μm.

**Fig. 1:**
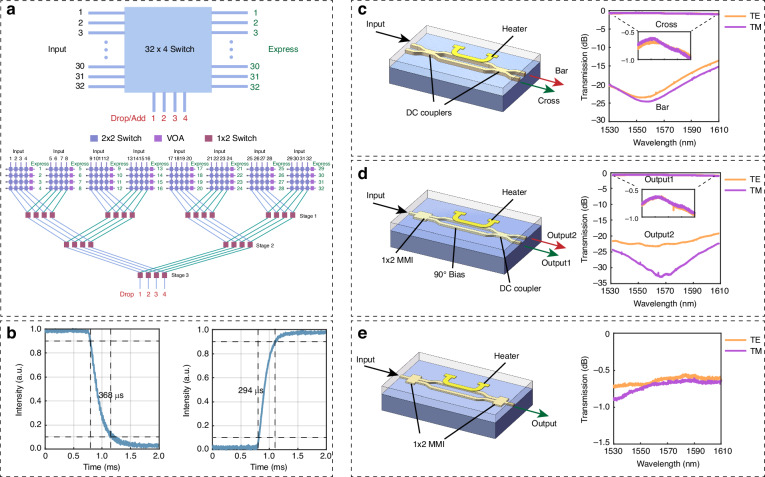
**The architecture of the 32** **×** **4 optical switch and the elements**. **a** The schematic and the topology of 32 × 4 switch. **b** The temporal response of the thermal-optical MZI switch. **c** The structure and optical response of MZI with 2 directional couplers when the heater is OFF. **d** The structure and optical response of MZI with directional coupler and MMI when the heater is OFF. **e** The structure and the optical response of MZI with two MMIs when the heater is OFF. Responses include fiber-to-chip coupling

The architecture of the 32 × 4 optical switch is illustrated in Fig. 1a. It features 4 drop channels, and the routing implementation is based on 4 × 4 and 1 × 4 matrices. The 4 × 4 matrix functions as a crossbar switch unit, determining the channels to be dropped^40^. When the heater is OFF, light passes through the cross port and connects with express channels. The bar port is linked with the corresponding 1 × 4 matrix, and the cascaded 1 × 4 matrices drop the target 4 channels to specific drop ports without conflict. This topology requires only 156 MZIs. VOAs are added after the cross port of each 4 × 4 crossbar unit to enable the ability of flatting the power of 32 express ports. Therefore, the total number of switches in the complete circuit is 188. The VOA can also be fully powered on when the corresponding port is dropped, in order to achieve low crosstalk at the express port (in which a new signal will be typically added). Even though the paths of different channels are not balanced with this topology, it will not cause large path-dependent IL differences, because of the low propagation loss of the waveguide and switch elements. Here, we have described the drop functionality. In transmission application, add and drop are always used in pairs, and the switch can used for both dropping and adding signals.

The basic element of the 4 × 4 matrix is a 2 × 2 switch, while the 1 × 4 matrix uses a 1 × 2 switch. The 2 × 2 switch comprises two 2 × 2 directional couplers and two balanced arms. The 1 × 2 switch replaces one directional coupler with a multi-mode interference (MMI) and adds an extra pi/2 bias on one arm. The VOA is based on a 1 × 1 switch, which replaces two directional couplers with two 1 × 2 MMIs. All switches have the heater on one arm to change the waveguide index, functioning as a phase shifter with the TO effect. Figure 1c–e depicts the transmission response of a single MZI switch element with different combinations of directional coupler and MMI when the heater is OFF. In Fig. 1c, light exits from the cross port when the heater is OFF, but it will be switched to the bar port when the heater is ON and receive a π phase shift. For Fig. 1d, the two arms already have a π/2 phase difference when the heater is OFF, so the light goes out from output1, when the heater is ON and shifts π, the optical response of output1 and output2 will switch with each other. In Fig. 1e, when heater is OFF, the light is transmitted from the output. After the heater is ON, and shifts π, the output has no power. The loss is within 1 dB over the C and L bands of transmitted ports for all of these three cases, including the coupling loss of two fiber-coupled facets. The coupling loss of two facets is as low as 0.5 dB at 1550 nm, indicating that the on-chip loss of a single MZI is very small, below 0.1 dB in the C band. Furthermore, the extinction ratio is higher than −20 dB over the full band. The response time was measured with a fully packaged switch device and recorded with an oscilloscope. Figure 1b shows the temporal response of the switch and the response time is 294 μs and 368 μs for rise or fall. The speed is sufficient for failure recovery applications and network reconfiguration^10,41^.

### Optical via design

**Fig. 2:**
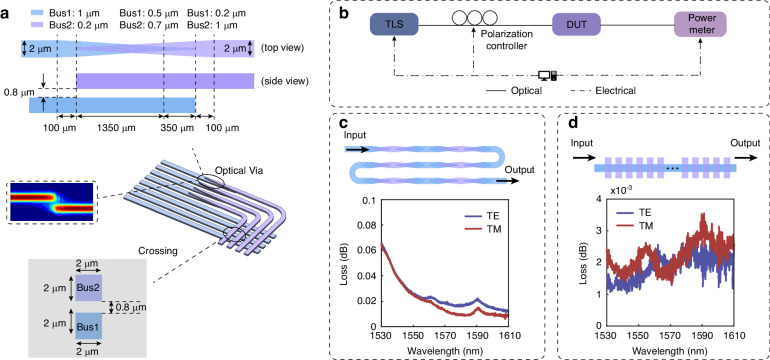
**The performance of optical via and optical crossing. a** The schematic of the optical via. **b** The measurement setup of the optical response. **c** The cascaded via and the optical spectrum of single via of both TE and TM modes. **d** The cascaded crossings of two layers and the optical response of a single crossing of both TE and TM modes

Considering the topology which consists of multiple crossings between the different channels. Direct in-plane crossings of waveguides would result in high excess loss and increase the crosstalk. To circumvent this, two bus layers are employed, one above the other in 3D. The two layers are identical and separated by a distance of 800 nm. Optical vias are used to achieve this^42^, as shown in the schematic in Fig. 2a. The thickness of the functional top and bottom layers is 2 μm and the gap is 800 nm. The vertical coupler goes through two stages. In the first stage, the bottom bus goes from 1 μm to 0.5 μm and the top bus from 0.2 μm to 0.7 μm over a 1350 μm long taper. In the second stage, the bottom bus is tapered down to 0.2 μm and the width of the top bus expands to 1 μm with a length of 350 μm. After the vertical coupler, the top bus tapers from 1 μm to 2 μm with a length of 100 μm. The schematic is shown in Fig. 2a. A test structure consisting of 30 cascaded vias (shown in the top part of Fig. 2c) was used to characterize the individual via loss and reduce measurement uncertainty. The measured loss of single via is depicted in the lower part of Fig. 2c, showing the loss is below 0.06 dB across C and L bands with PDL below 0.01 dB. To evaluate the cross-talk of two layers with a 800 nm gap, 48 crossings are also made in the test structure, and the loss of a single crossing is lower than 0.003 dB over the C and L bands, as shown in Fig. 2d. The measurements of the test structures were conducted using the setup shown in Fig. 2b. The input and output of the optical waveguides are aligned to the input and output optical fibers on the alignment stage. A tunable laser source covering C and L bands is connected to a polarization controller and used to launch the signal into the waveguide through the input fiber. The output fiber is connected to the power meter to measure the power after transmission, where losses can be directly obtained after subtracting the fiber-fiber reference.

### Express ports performance

**Fig. 3:**
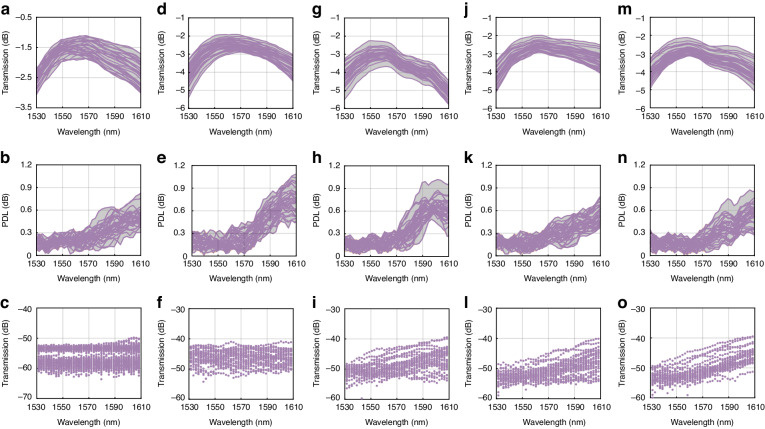
**The fiber-to-fiber loss, PDL, and crosstalk of 32** **×** **4 optical switch. a** The loss of 32 input channels to corresponding express ports. **b** The PDL of 32 input channels corresponding to express ports. **c** The crosstalk between express and drop 1,2,3,4. **d** The loss of 32 input channels to drop channel 1. **e** The PDL of 32 input channels to drop channel 1. **f** The crosstalk between drop 1 and corresponding express. **g** The loss of 32 input channels to drop channel 2. **h** The PDL of 32 input channels to drop channel 2. **i** The crosstalk between drop 2 and corresponding express. **j** The loss of 32 input channels to drop channel 3. **k** The PDL of 32 input channels to drop channel 3. **l** The crosstalk between drop 3 and corresponding express. **m** The loss of 32 input channels to drop channel 4. **n** The PDL of 32 input channels to drop channel 4. **o** The crosstalk between drop 4 and corresponding express

The default (un-powered) state of the 32 × 4 optical switch has all 32 input channels routed to the express ports without any tuning of MZI. Each channel goes through five switch elements and all the express channels are rotated back to the same side of the input channels (single-sided packaging). The fiber-to-fiber loss was measured and depicted in Fig. 3a with purple lines, including the two facets coupling loss between SMF and HDSG edge coupler, propagation loss, the loss of switch elements, and alignment loss of the very large 128-channel fiber array. The average losses of transverse electric (TE) and transverse magnetic (TM) mode of 32 channels are below 3 dB across C and L bands and below 2 dB at 1550 nm. The grey area indicates the lowest and highest loss of the 32 channels, and the loss variation of 32 channels is below 1.28 dB over the C and L bands. From Fig. 3b, it can be observed that the PDL is lower than 0.3 dB at the C band for all express ports. The power of four drop ports is monitored while measuring the express loss. The crosstalk is plotted in Fig. 3c, which is below -50 dB for all 4 ports across C and L bands.

### Drop/Add ports performance

Figure 3d, g, j and m show the fiber-to-fiber losses of each input port to drop 1, drop 2, drop 3, and drop 4, respectively. The losses of all cases are below 5 dB across C and L bands, and the loss is below 3.5 dB at 1550 nm. For the 32 channels of all the cases of drop 1, drop 2, drop 3, and drop 4, the loss variations are within 2.2 dB over C and L bands, mainly induced by the number of MZIs in the path (ranging from 7 to 10), the path length, and the coupling variation. The crosstalk to the corresponding express port is measured at the same time as the measurement of each drop port loss. The crosstalk is lower than -40 dB in the C band for all the cases, which are depicted in Fig. 3f, i, l and o. Figure 3e, h, k, and n are the PDL of all cases, and the PDL is within 0.3 dB in the C band, but higher in the L band because the MZIs and traces were optimized at the C band in this device.

**Fig. 4:**
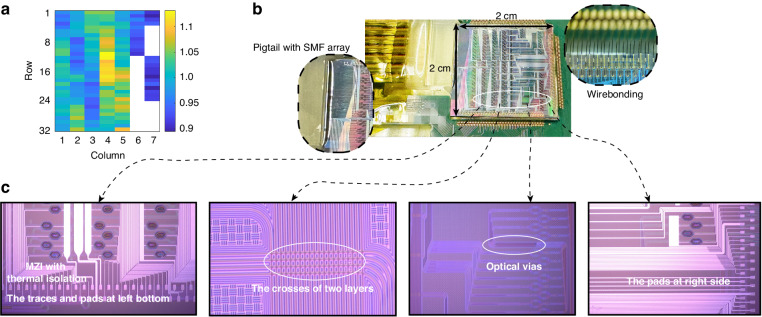
**The power consumption map, the package, and the microscope images of 32** **×** **4 optical switch. a** The map of normalized power consumption of the switches referenced to the overall average (donated as value 1.0). **b** The packaged 32x4 switch. **c** The microscope images of different structures

The drop port responses were measured with full optical and electronic packaging at room temperature without a temperature controller. For optical packaging, we used a 128-channel SMF array edge coupled to the chip. For electronics, we mounted the chip on a ceramic substrate glued to the printed circuit board (PCB), then wire-bonded the heater pads of the chip with the corresponding pads on the PCB, as shown in Fig. 4b. When measuring the drop loss, the corresponding heaters need to be powered on to drop the selected input channels to the target drop ports. To optimize drop ports’ performances, all the switches are scanned to find the optimum power needed to achieve minimal drop loss. For our device, the heater is tungsten, and the metal traces are aluminum (described in the Method section). The scanned optimized power here includes the heater power and the power dissipated on the aluminum traces. The power consumption of each switch is collected and the average power consumption of 188 switches is about 130 mW per switch. We use the average value to normalize the power consumption of each switch and map the heater power according to the real layout, which is show in Fig. 4a. Each grid here represents one switch and the blank grid means there is no switch. The color from blue to yellow corresponds to power consumption ranging from low to high. The variation of power consumption over 188 switches is about ±10% based on average. The power consumption is higher in the middle of the chip because of the switches are further from the edge and have longer metal traces with higher trace resistance. When taking the trace resistance into account, the result shows that the switches are very uniform within the 2 cm × 2 cm chip area.

### Wavelength selective switch performance

Figure 3 shows the measured fiber-to-fiber IL, PDL, and crosstalk of the fabricated 32 × 4 optical switch. The colorless switch exhibits low IL across the C and L bands, making it compatible with broadband ROADM applications having any dense wavelength division multiplexing (DWDM) channel spacing and international telecommunication union (ITU) grid requirements. However, even broadband MZIs have a certain amount of wavelength dependence upon tuning due to the wavelength-dependent differential phase shift. When each input channel corresponds to a specific wavelength, the corresponding MZIs can be more precisely optimized to that wavelength, resulting in improved performance in a wavelength-selective application.

**Fig. 5:**
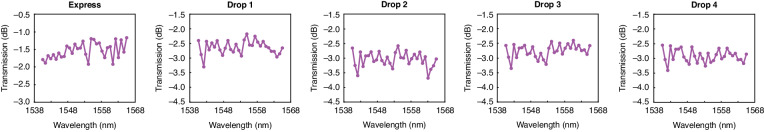
**The extracted fiber-to-fiber loss of the switch aligned with 100** **GHz DWDM according to the ITU grid**

To qualify the performance of the switch when it is connected to a 100 GHz DWDM to work as 100 GHz ROADM. We assigned the wavelengths from 1540.56 nm to 1565.5 nm according to the ITU grid to channels 1 to 32 of the switch and extracted the fiber-to-fiber loss of all cases, presented in Fig. 5. The fiber-to-fiber losses of 32 channels in the express case are below 2 dB, and in drop cases, the losses are lower than 3.5 dB.

### VOAs performance

**Fig. 6:**
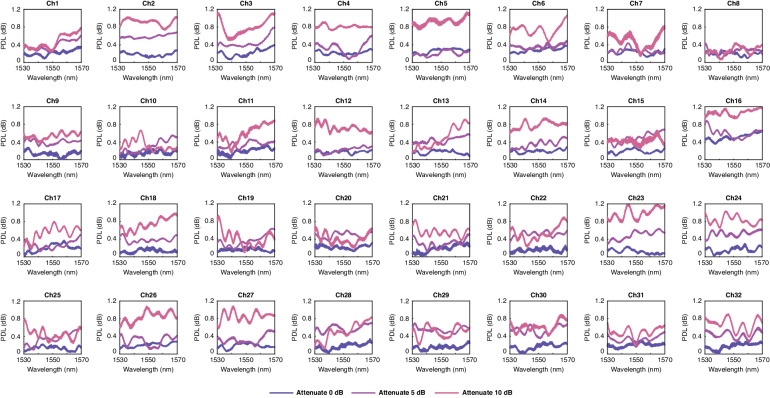
**The PDL of 32 VOAs with settings of 0** **dB attenuation, 5** **dB attenuation, and 10** **dB attenuation**

To expand the functionality of the 32 × 4 switch, we also added VOAs at each express channel to allow the attenuation of the express power. The power can be tuned within a range of 0-20 dB, but the key to evaluate the performance of the VOA is the PDL at high attenuation because the attenuation can degrade the PDL performance. Here, we measured two separate cases, 5 dB attenuation and 10 dB attenuation of 32 VOAs, presented in Fig. 6. For 5 dB attenuation, all the 32 VOA can maintain total circuit PDL lower than 0.6 dB, and for 10 dB attenuation, the total circuit PDL still remains within 1.0 dB.

### Transmission performance

**Fig. 7:**
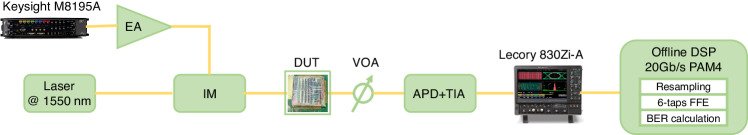
**The schematic of the BER measurement setup**

To verify the performance of the 32 × 4 switch in the transmission system, we conducted a 20 Gb/s PAM-4 transmission experiment. Figure 7 shows the intensity modulation/direct detection (IM/DD) optical transmission system schematic. The pseudo-random binary sequence (PRBS: 2^15^-1) signals in this setup are generated by the arbitrary waveform generator (Keysight M8195A) at the sampling rate of 54 GSa/s. These signals are then modulated by a 10 G-class intensity modulator (IM) at the 1550 nm wavelength before being launched into the SMF. The power of the modulated optical signals fed into the 32 × 4 optical switch is set to around −1.0 dBm, with the power at the output of the switch properly attenuated by a VOA before being detected by the 10 G-class avalanche photodiode-transfer-impedance-amplifier (APD-TIA). The detected signals are then sampled by the oscilloscope (LeCory 830Zi-A) at the sampling rate of 80 GSa/s, where the offline digital signal processing (DSP) is employed to form a light equalization and the Bit-error-rate (BER) calculation.

**Fig. 8:**
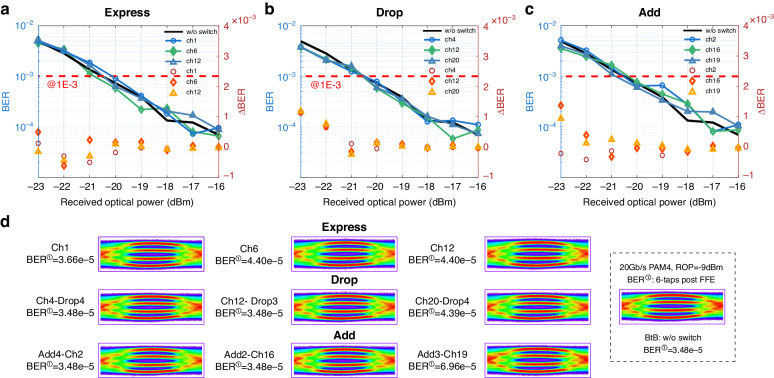
**The BER results and eye diagrams of 32** **×** **4 optical switch**. **a** The BER results of selected express channels. **b** The BER results of selected drop channels. **c** The BER results of selected add channels. **d** The eye diagram of the corresponding express, drop, and add cases

Multiple channels of the switch are randomly selected to measure the express, add, and drop functions. Their performances are evaluated at the receiver side by the eye diagrams before the DSP, and the calculated BER after the 6-tap feedforward equalisation (FFE) DSP. According to the BER results depicted in Fig. 8a–c, it can be observed that compared to the system without the switch, the system employed with the proposed switch suffers little degradations in terms of expressing, adding, and dropping the transmitted optical signals. The calculated BER differences at multiple received optical power (ROP) between the two cases are lower than 10^−4^ with multiple measurements. Meanwhile, Fig. 8d also shows the eye diagrams of the express, add, and drop performances at a specific ROP of -9 dBm, indicating the performances are comparable with the ones without the switch in the system.

## Discussion

Extensive progress has been made in the development of optical switches over the past few decades, spanning a range of applications and featuring various topologies and packaging solutions. One specific application that stands out is ROADMs, valued for its scalability and adaptability. Integrated optical MZI-based ROADMs offer a robust, scalable, solid-state solution that can be manufactured in volume. Additionally, they hold the potential for integration of other elements such as WDM filters, and VOAs.

However, the current integrated optical approach faces challenges at the two extremes of the index contrast spectrum. On the one hand, achieving compact and large-scale switch arrays based on conventional low-index PLCs is difficult to achieve because of the large waveguide bend radius requirement. On the other hand, very high index waveguides such as Si and SiN struggle with high waveguide loss, PDL, polarization-dependent wavelength (PDW) sensitivity, significant fiber coupling loss, and inconsistent fabrication quality and uniformity, making them less likely to meet the commercial requirement.

To strike a balance between performance, footprint, and commercial manufacturability, a moderately high index contrast of 10% is employed. This choice allows for the creation of compact structures, while simultaneously ensuring that the loss, PDL, PDW, and fiber coupling remain markedly superior to those of the Si and SiN platforms. Table 1 shows a comparison of various high-index contrast-based TO switch matrices. In comparison to the HDSG switches, the Si and SiN switches have faster-switching speed and a smaller footprint but with a substantially higher IL, PDL and crosstalk. This makes the Si and SiN switches less equipped for commercial ROADM deployment. Additionally, most of the Si and SiN switches of consideration are for symmetrical switch matrices, but the architecture presented here also uses a more efficient unsymmetrical topology, reducing the number of switch elements from 256 to 188 compared to the topology of using four parallel 32 × 1 blocks. For large-scale switches, the on-chip loss and cross talk not only depends on the loss of each switch element, but it also depends on the loss of crossings between the waveguides. Utilizing a 3D integration process, ultra-low loss crossings (below 0.003 dB) are achieved by distributing waveguide crossings in two separate bus layers and with identical waveguide dimensions. Apart from lowering the loss and crosstalk, this process can also extend the integration dimension in the vertical direction since both layers support functional elements. Owing to the moderately confined refractive index of our waveguide platform (~10%), tight bend radii can be achieved, yet the mode is not so tightly confined such as in SiN or Si, that efficient broadband spot size converters for matching standard SMF can be realized. The measured coupling loss between SMF and the waveguides is about 0.5 dB at 1550 nm and is below 0.8 dB across the C + L bands for two facets. This enables the fiber-to-fiber loss of the 32 × 4 circuit to be within 5 dB over the entire 80 nm bandwidth of C + L bands. Moreover, the moderately high index contrast allows designing the waveguide to support both polarization of TE and TM mode propagation to achieve low PDL. For the fabricated switch device, the PDL of all express and drop/add cases are below 0.3 dB in the C band, which is vital to the telecom application as well as other applications like quantum communication, photonic neural network, etc.

**Table 1:** The performance comparison of the TO switches

Work	Material	Structure	^a^IL (dB)	MZI number per path	Cross-talk (dB)	Coupling method	PDL (dB)	Speed (μs)	Footprint (cm × cm)
^23^	Si (220 nm)	8 × 8	<11.8 C band	6	<−30	Edge coupling (SiO2 cantilever)	TE only	250	0.8 × 0.8
^10^	Si (1.5 μm)	8 × 8	6 @1570 nm	Max: 10 min: 8	<−35	Edge coupling (narrow-core fiber)	<1.5 dB @ 1570 nm	15	1.2 × 1.4
^38^	Si	16 ×16	16.2 @1560 nm	7	<−35	Vertical coupling (grating coupler)	TE only	22	0.7 × 0.36
^22^	Si (225 nm)	32 × 32	10.8 @1547 nm	32	<−20	Edge coupling (extremely high-Δ silica PLC+ high-Δ fiber)	TE only	30	1 × 2.6
^[b31^	Si/SiN	32 × 32	<15.54 @1580 nm	10	<−35	Edge coupling (PMF)	TE only	388.1	2.4 × 1.8
**This work**	**HDSG**	**32 × 4**	**Express:** < **3** **Drop:** < **5** **(C** + **L bands)**	**Express: 5** **Drop max: 10** **Drop min: 7**	**Express:<−50** **Drop:<−40**	**Edge coupling** **(SMF)**	**0.3** **(C band)**	**368**	**2 × 2**

^a^IL here is the sum of coupling loss and on-chip loss;

^b^Only take with undercut case of this work.

In conclusion, we demonstrate a large-scale switch for ROADM application, which immediately paves the way for the commercial usage of optical switches. With the low loss and polarization insensitive capability, more sophisticated TO switch circuits can be achieved in future advanced applications.

## Materials and methods

### Simulation of optical via and crossing

The beam propagation method from RSoft^@^ is used for the simulation of the loss of optical via and waveguide crossings. A total of 15 wavelength points are selected from 1530 nm to 1590 nm to run the simulation to obtain the optimized broadband response. The detailed parameters are depicted in the Optical via Design section.

### Device fabrication

The entire fabrication process is carried out in a CMOS foundry. The process starts with silicon wafers having 6 μm native thermal oxide (Tox) acting as the lower cladding. HDSG core film is deposited with PECVD, forming the bottom bus waveguide, which is then patterned using standard I-line lithography and clad with oxide. The wafers undergo a chemical mechanical polishing (CMP) step to achieve a flat topography before deposition of the second waveguide layer. Tungsten heaters and aluminum traces are deposited next and a final oxide cladding covers the entire wafer. A deep etch is applied to MZIs to alleviate the thermal crosstalk. During the process, fiber-matched mode expanders are monolithically grown to expand the 2 μm × 2 μm waveguide mode field up to the 10.4 μm × 10.4 μm SMF mode field.

### Device packaging

The device is pigtailed with 128-channels single-mode fiber array with an edge coupling strategy. Then the pigtailed device is mounted on the PCB bond with silver paste. The pads of the device are connected with PCB through wire bonding. The switches are driven by multiple current sources and the maximum current of each channel can go 120 mA. The current can be adjusted through registers with a resolution of 16 bits. All the sources are controlled by microcontroller unit through the serial peripheral interface (SPI).

### Switch configuration for dropping input ports

The MZIs are labeled from No. 1 to No. 188 from left to right and top to bottom as depicted in the schematics in Fig. 1a. For all the switches in the 1 × 4 matrices, the left path is transmitted, and the right path is blocked when the heater is OFF. Therefore, to drop input 1 to port 1, switch No. 1 must be powered ON, and to get better crosstalk, switch NO.5 (the VOA) will also need to be powered ON. This is the case with the minimum number of switches that need to be heated. When input 32 needs to be dropped to port 4, switch No.159, No.176, No.184, No.188 and NO.160 (the VOA) should be powered ON. This path requires the maximum number of switches to be turned on. If 4 drop ports work together, the total maximum number of switches that are powered on at the same time will be 20, with a power consumption within 2.6 W.
